# Comparison of Sysmex XN-V body fluid mode and deep-learning-based quantification with manual techniques for total nucleated cell count and differential count for equine bronchoalveolar lavage samples

**DOI:** 10.1186/s12917-024-03884-5

**Published:** 2024-02-05

**Authors:** Sandra Lapsina, Barbara Riond, Regina Hofmann-Lehmann, Martina Stirn

**Affiliations:** Clinical Laboratory, Department of Clinical Diagnostics and Services, Vetsuisse Faculty, University of Zurich, Winterthurerstrasse 260, CH-8057 Zurich, Switzerland

**Keywords:** Equine, Bronchoalveolar lavage, Sysmex, Body fluid mode, Regating, Total nucleated cell count, Differential count, Deep-learning, Artificial intelligence

## Abstract

**Background:**

Bronchoalveolar lavage (BAL) is a diagnostic method for the assessment of the lower respiratory airway health status in horses. Differential cell count and sometimes also total nucleated cell count (TNCC) are routinely measured by time-consuming manual methods, while faster automated methods exist. The aims of this study were to compare: 1) the Sysmex XN-V body fluid (BF) mode with the manual techniques for TNCC and two-part differential into mononuclear and polymorphonuclear cells; 2) the Olympus VS200 slide scanner and software generated deep-learning-based algorithm with manual techniques for four-part differential cell count into alveolar macrophages, lymphocytes, neutrophils, and mast cells. The methods were compared in 69 clinical BAL samples.

**Results:**

Incorrect gating by the Sysmex BF mode was observed on many scattergrams, therefore all samples were reanalyzed with manually set gates. For the TNCC, a proportional and systematic bias with a correlation of *r* = 0.79 was seen when comparing the Sysmex BF mode with manual methods. For the two-part differential count, a mild constant and proportional bias and a very small mean difference with moderate limits of agreement with a correlation of *r* = 0.84 and 0.83 were seen when comparing the Sysmex BF mode with manual methods. The Sysmex BF mode classified significantly more samples as abnormal based on the TNCC and the two-part differential compared to the manual method. When comparing the Olympus VS200 deep-learning-based algorithm with manual methods for the four-part differential cell count, a very small bias in the regression analysis and a very small mean difference in the difference plot, as well as a correlation of *r* = 0.85 to 0.92 were observed for all four cell categories. The Olympus VS200 deep-learning-based algorithm also showed better precision than manual methods for the four-part differential cell count, especially with an increasing number of analyzed cells.

**Conclusions:**

The Sysmex XN-V BF mode can be used for TNCC and two-part differential count measurements after reanalyzing the samples with manually set gates. The Olympus VS200 deep-learning-based algorithm correlates well with the manual methods, while showing better precision and can be used for a four-part differential cell count.

**Supplementary Information:**

The online version contains supplementary material available at 10.1186/s12917-024-03884-5.

## Background

Bronchoalveolar lavage (BAL) is a diagnostic method for the assessment of the lower respiratory airway health status in various animal species, including horses. It is performed by infusing a quantified volume of fluid in the lungs via a bronchoscope or a BAL catheter and then aspirating it for assessment. In horses, the evaluation of BAL fluid samples is considered most useful for diagnosing diffuse pulmonary diseases such as the equine asthma syndrome, exercise-induced pulmonary hemorrhage, and certain infectious diseases (e.g., equine herpesvirus-5) [1, 2].

The total nucleated cell count (TNCC) and the differential cell count are two parameters routinely determined in BAL fluid samples. TNCC is a controversial parameter in literature since it varies considerably based on the lavage volume introduced in and recovered from the lungs. However, TNCC provides some useful information in case of strictly standardized BAL protocols and is also used in research [3, 4]. Depending on the protocol of each laboratory TNCC can either include or exclude columnar epithelial cells [1, 5, 6]. The gold standard for TNCC measurement is manual counting with a hemocytometer, and the cut-off value for TNCC without columnar epithelial cells in an unremarkable sample is considered < 530 cells/µL when 250 mL sodium chloride has been infused. Aspiration is expected to yield 50 to 70% of the fluid volume infused, moreover the retrieved volume tends to negatively correlate with the presence and severity of an inflammation as it may cause bronchoconstriction or even an airway collapse thus hindering an effective aspiration [5, 7].

The differential cell count is considered to be a more reliable parameter than TNCC for the diagnostic workup of respiratory diseases since it does not depend on the volume of saline solution used for lavage. Alveolar macrophages, lymphocytes, neutrophils, eosinophils, and mast cells are differentiated on cytospin preparations via light microscopy as the gold standard. Some laboratories also include epithelial cells within the differential cell count [1, 4, 8]. Since the cells are not evenly distributed several hundred cells need to be counted for an accurate and precise differential cell count: a differential cell count of 200 cells is considered reproducible for alveolar macrophages, neutrophils, and eosinophils while to reach adequate reproducibility in all cell types counting of at least 500 cells is advisable [9].

Counting with a hemocytometer and light microscopy is a time-consuming and tiresome manual method, which additionally has considerable inter- and intraobserver variability and requires skilled personnel [10, 11]. Due to this reason, automatization of cell quantification and differentiation in BAL fluids is desirable.

Regarding TNCC and differential cell count in BAL fluid by automated cell counters, only few studies are available. In human medicine, the Coulter® counter of electrical sensing zone method demonstrated a correlation of *r* = 0.81 to 0.84 to manual methods and better repeatability for automated TNCC measurement in a study from 1994, while older studies claim either underestimation or overestimation of the cell count by Coulter® counters [12–16]. Meanwhile in veterinary medicine, a study of pulmonary toxicology in BAL of mice and rats is available [17]. Measurements of three different TNCC counting methods—hemocytometer, impedance counter Coulter Multisizer III and flow cytometer FACSCalibur employing beads and CD45 antibodies – were obtained and compared after the animals had undergone inhalation or intratracheal instillation of pulmonary toxicants. Correlation was generally observed between the three methods, with better correlation seen in samples with less cytotoxicity, indicated by lower measured lactate dehydrogenase levels in BAL fluid. In more cytotoxic samples the Coulter Multisizer III showed higher TNCC than the flow cytometry method which was explained through the erroneous counting of necrotic cellular debris as viable cells by the Coulter Multisizer III analyzer [17]. Another study from rats and mice provided excellent agreement for manual and automated TNCC using Sysmex and ADVIA hematology analyzer in blood mode. Both instruments use flow cytometry and the Sysmex analyzer provided similar percentages in four-part differential count obtained by manually set gates when compared to manual counts [6].

During the last fifteen years, some hematology analyzers have become equipped with a special body fluid (BF) mode, starting with the Sysmex XE—5000 in 2007 [18]. The Sysmex BF offers the measurements of both total nucleated cell count (TC-BF; further in the text referred to as BF-TNCC) and white blood cell count (WBC-BF; further in the text referred to as BF-WBC). The difference between these two parameters is that the BF-WBC recognizes and counts only leukocytes based on their specific cellular properties of nucleic acid fluorescence and cell granularity, while the BF-TNCC in addition to the leukocytes also counts large and highly fluorescent cells such as mesothelial cells [19]. To our knowledge no information is available on whether alveolar macrophages and columnar epithelial cells of equine BAL would be included in either of these counts, however, as the alveolar macrophages visually resemble mononuclear (MN) cells found in other BFs, it can be assumed they would be included in both BF-WBC and BF-TNCC, while the columnar epithelial cells would probably be included only in BF-TNCC. Additionally, a two-part differentiation into MN and polymorphonuclear (PMN) cells using flow cytometry is performed. It is possible to set manual gates thus creating different profiles on this analyzer. BF mode analyzes a larger sample volume thus subsequently counting about three times more cells than the whole blood mode [20]. Studies with BF mode on Sysmex XE and Sysmex XN analyzers have shown excellent correlations for the TNCC or WBC count with the manual methods for peritoneal, pleural, synovial, and cerebrospinal fluids (CSF) in human samples [21, 22]. To our knowledge, no study has investigated the BF mode in BAL fluid samples in either human or veterinary medicine.

Another highly promising and rapidly developing field is artificial intelligence (AI) with its branch of machine learning which mimics the problem-solving and decision-making abilities of the human mind. Deep-learning is a subdivision of machine learning and is based on artificial neuronal networks—algorithms that automatically generate identifying characteristics from the processed specimens. Deep-learning is increasingly used in cytology since it allows to process large quantities of data in a relatively short period of time and can be operated by technical personnel without medical knowledge. In case of the latter, the evaluation for possible errors and detection of findings that the AI is not trained for takes place in a subsequent separate step of medical validation and is performed by a person with the appropriate medical knowledge. In human medicine, machine learning and deep-learning have been used for neoplastic cell detection and differentiation between malignant and benign processes in a wide variety of solid tissues and some BFs [23, 24]. In a study from 2021, deep-learning technology also managed to successfully identify the majority of cells in BAL fluid from human patients with respiratory symptoms [25]. In veterinary medicine, a study of pulmonary hemosiderophage detection on cytospin preparations in horses by both manual method and deep-learning algorithm was performed [26]. In this study, a concordance with ground truth data (a term referring to real world data used in training of an algorithm) was assessed for both methods and the deep-learning algorithm partially outperformed the manual detection by showing a concordance of 85% whereas the manual detection reached 66 to 86% [26]. In a study of bovine endometrial cytology, a moderate to substantial agreement was obtained between the manual count via light microscopy and the deep-learning technology using Oculyze Monitoring Uterine Health system for detection of PMN cells once the threshold was set to > 10% PMN cells [27].

We hypothesize that it is possible to obtain accurate automated measurements for both TNCC and differential cell count in BAL fluid samples. We also hypothesize that the automated methods will be more precise than the manual methods since more cells can be counted with them. Therefore, the aims of this prospective study were:

1. To compare the Sysmex XN-V BF mode with manual techniques for TNCC and two-part differential cell count in BAL samples,
2. To compare the Olympus VS200 software generated deep-learning based algorithm with manual technique on digital images of scanned BAL cytospins for four-part differential cell count (Table 1).

**Table 1:** Table of study design showing the measured parameters and the compared methods for each parameter

Parameters	TNCC	Two-part differential cell count	Four-part differential cell count
**Methods**			
Sysmex XN-V BF mode (automated counting)	69 BAL samples	69 BAL samples	
Olympus VS200 scanner and software a) Manual differentiation b) Automated differentiation using AI algorithm			68 BAL cytospins, digital images of 200 cells each
Light microscopy (manual counting)	69 BAL samples, Neubauer chamber	69 BAL cytospins	69 BAL cytospins^a^

*AI* Artificial intelligence, *BF* Body fluid, *BAL* Bronchoalveolar lavage, *TNCC* Total nucleated cell count

^a^Only for classification of samples according to ACVIM Consensus Statement and the precision study

## Results

### Sysmex XN-V BF mode versus manual methods: TNCC

**Fig. 1:**
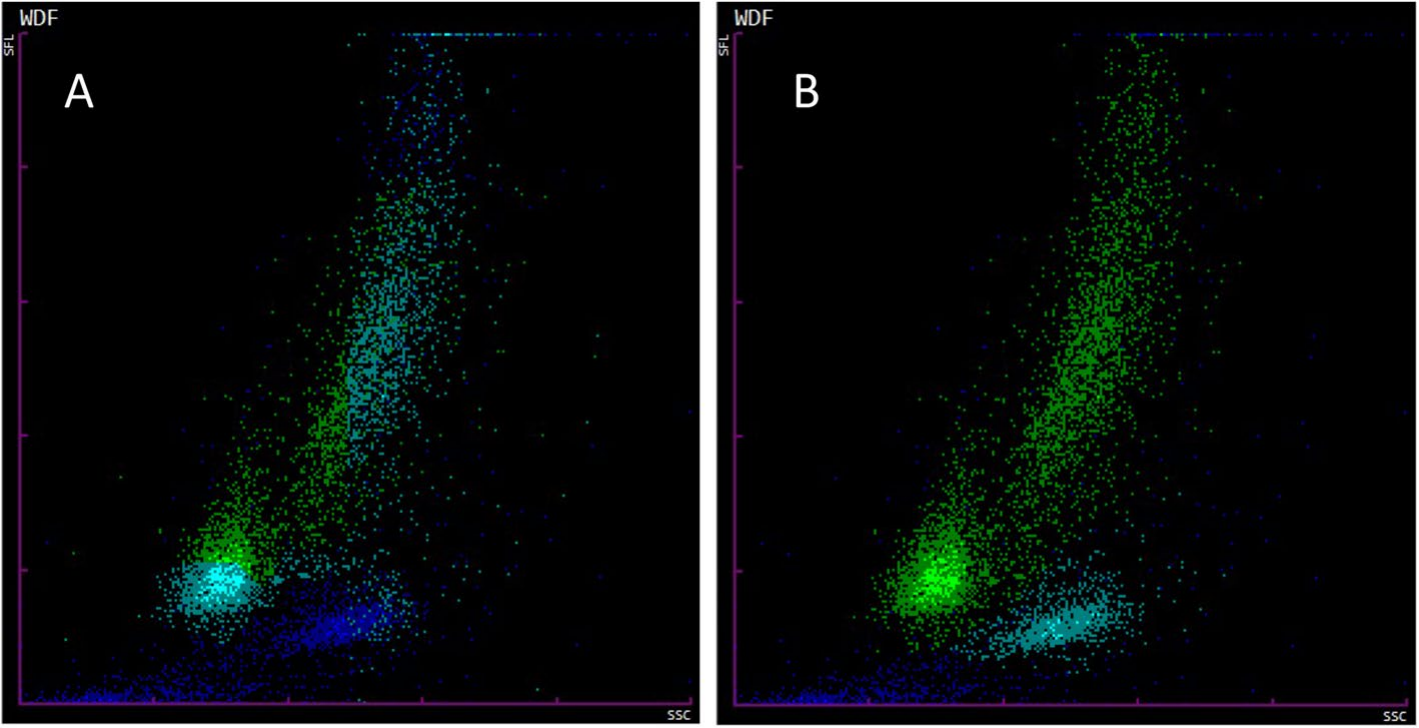
Comparison of a representative Sysmex XN-V BF mode scattergram before (**A**) and after (**B**) setting of manual gates. Debris is depicted as dark blue, MN cells as green, and PMN cells as light blue. Before manual regating (**A**) most of the PMN cells are classified as debris, while MN cells are partly counted as debris, MN cells, and PMN cells. After manual regating (**B**) the cell populations are correctly distinguished

TNCC were obtained using the Sysmex XN-V BF mode as well as manual counting via light microscopy. The Sysmex BF mode allows classification of the events into debris, MN or PMN cells by separating these entities by color. When analyzed with the Sysmex BF mode an incorrect automated gating for both MN and PMN cells was observed on the scattergrams in many BAL samples (for an example see Fig. 1A; after manual regating see Fig. 1B; for the depiction of the manually set gates see Additional file 1A and B). The distinctly demarcated cell populations were often misidentified on the scattergram, classifying PMN cells as debris, while MN cells were depicted as a mixture of debris, MN and PMN cells. To solve this issue and prior to including samples for the comparison study a manual gate was established on one BAL sample in the extended scattergram with clearly visible, but misclassified PMN and MN cells (Additional file 1B). This manual gate was then applied to all other samples while carefully assessing its adequacy for each sample. The established manual gate fit all samples according to visual inspection of the scattergrams. The results prior to and after regating were significantly different (*p*-value < 0.001). The raw data for all measurements are provided as Additional file 2, while the results before regating are provided as Additional files 3 and 4.

**Fig. 2:**
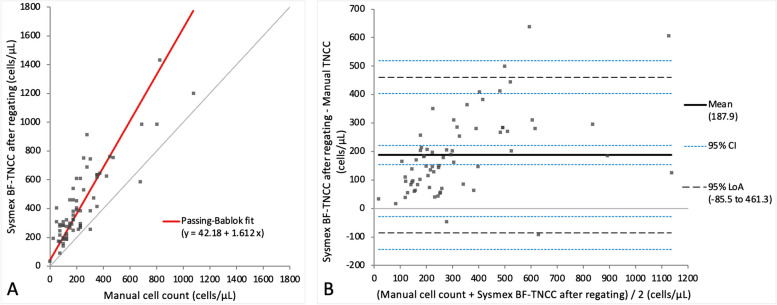
Agreement between the manual TNCC and Sysmex BF-TNCC (cells/µL) after regating. The graph on the left (**A**) is a Passing-Bablok regression analysis with intercept 42.18 (-14.50 to 106.30)* and slope 1.61 (1.33 to 2.04)*. The graph on the right (**B**) is a Bland–Altman difference plot. The thin horizontal grey line (0 at the y-axis) is the line of identity, and the thick black line indicates the bias (mean difference between methods), with its confidence intervals as thin blue dashed lines. The black dashed horizontal lines are the 95% limits of agreement with their 95% confidence intervals as the thin blue dashed lines. The mean difference is 187.9 (154.37 to 221.40)* cells/µL, the Lower Limit of Agreement is -85.5 (-143.08 to -27.98)* cells/µL, the Upper Limit of Agreement is 461.3 (403.75 to 518.85)* cells/µL. *Numbers in parentheses are 95% confidence intervals

**Fig. 3:**
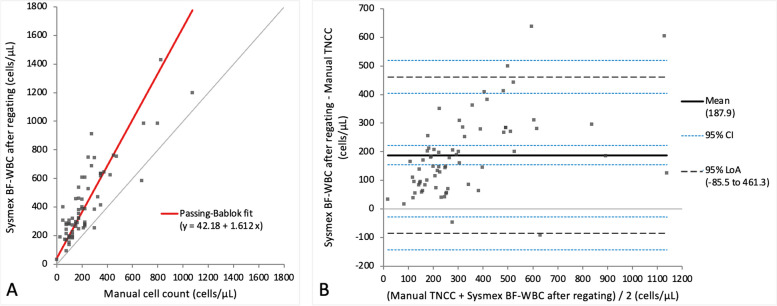
Agreement between the manual TNCC and Sysmex BF-WBC (cells/µL) after regating. The graph on the left (**A**) is a Passing-Bablok regression analysis with intercept 42.18 (-11.30 to 106.00)* and slope 1.61 (1.31 to 2.02)*. The graph on the right (**B**) is a Bland–Altman difference plot. The thin horizontal grey line (0 at the y-axis) is the line of identity, and the thick black line indicates the bias (mean difference between methods), with its confidence intervals as thin blue dashed lines. The black dashed horizontal lines are the 95% limits of agreement with their 95% confidence intervals as the thin blue dashed lines. The mean difference is 187.9 (154.37 to 221.40)* cells/µL, the Lower Limit of Agreement is -85.5 (-143.08 to -27.98)* cells/µL, the Upper Limit of Agreement is 461.3 (403.75 to 518.85)* cells/µL. *Numbers in parentheses are 95% confidence intervals

Method comparison results between the Sysmex BF mode and manual TNCC measurements before and after regating are depicted in Table 2 as well as Additional files 3 and 4 for results before regating and Figs. 2 and 3 for results after regating. BF-TNCC and BF-WBC yielded very similar values without any statistically significant difference after regating, therefore only BF-TNCC is further reported herein (Figs. 2A and B and Additional file 3A and B). Comparison of Sysmex BF-TNCC with manually obtained TNCC both before and after regating revealed a proportional and systematic TNCC bias on Passing-Bablok regression analysis. Regarding the Bland–Altman difference plot the regating slightly increased the mean bias, which reached 187.9 cells/µL after regating, slightly narrowed the limits of agreement and improved individual outliers (Fig. 2A and B). A correlation of *r* = 0.79 was observed between these two methods both before and after regating. There was a statistically significant difference between the TNCC measurements of both methods both before and after regating (*p*-value < 0.001). The BF-TNCC was significantly higher than the TNCC acquired manually (*p*-value < 0.001).

**Table 2:** Comparison of the TNCC and two-part differential cell count results from the Sysmex XN-V BF mode with those from the manual method

Parameter	Passing-Bablok regression analysis (95% CI Slope and Intercept)	Bias Bland–Altman difference plot (95% CI)	r	*p*-value
BF-TNCC before regating	y = 58 + 1.55x (Slope: 1.23 to 1.88 Intercept:—8.66 to 107.8)	171.3 cells/µL (136.44 to 206.33)	0.79	< 0.001
BF-TNCC after regating	y = 42.18 + 1.61x (Slope: 1.33 to 2.04 Intercept:—14.50 to 106.3)	187.9 cells/µL (154.37 to 221.40)	0.79	< 0.001
BF-WBC before regating	y = 39 + 1.48x (Slope: 1.67 to 1.83 Intercept: -19.75 to 93.00)	138.5 cells/µL (102.22 to 174.82)	0.78	< 0.001
BF-WBC after regating	y = 42.18 + 1.61x (Slope: 1.61 to 2.02 Intercept: -11.30 to 106.00)	187.9 cells/µL (154.37 to 221.40)	0.79	< 0.001
BF-MN% before regating	N	- 48.05% (- 54.31 to—41.79)	- 0.33	< 0.001
BF-MN% after regating	y = 15.39 + 0.83x (Slope: 0.65 to 0.94 Intercept: 4.36 to 32.68)	0.90% (-1.09 to 2.89)	0.84	0.92
BF-PMN% before regating	N	48.00% (41.76 to 54.25)	- 0.32	< 0.001
BF-PMN% after regating	y = 2.039 + 0.83x (Slope: 0.66 to 0.94 Intercept: 1.06 to 3.16)	- 0.94% (- 2.94 to 1.06)	0.83	0.88

*BF* Body fluid, *CI* Confidence interval, *MN* Mononuclear, *N* Noncalculable, *PMN* Polymorphonuclear, *p-value* Wilcoxon signed-rank test, *r* Spearman’s rank correlation coefficient, *TNCC* Total nucleated cell count, *WBC* White blood cell

Regarding the TNCC cut-off of < 530 cells/µL for unremarkable BAL samples as stated in the American College of Veterinary Internal Medicine (ACVIM) Consensus Statement from 2016 [5] 19/69 (27.5%) samples could be classified as abnormal by BF-TNCC after regating and 5/69 (7.3%) by manual TNCC (Table 3). Thus, the Sysmex BF mode recognized significantly more samples as abnormal than the manual method (*p*-value Fisher = 0.0029).

**Table 3:** Classification of samples by TNCC as unremarkable or abnormal according to the ACVIM Consensus Statement

**Parameter**	**Number of unremarkable samples** (TNCC < 530 cells/µL)	**Number of abnormal samples** (TNCC ≥ 530 cells/µL)
BF-TNCC after regating	50	19
BF-WBC after regating	50	19
Manual TNCC	64	5

*BF* Body fluid, *TNCC* Total nucleated cell count, *WBC* White blood cell

### Sysmex XN-V BF mode versus manual methods: two-part differential cell count

To compare the Sysmex BF mode and the manual two-part differential cell count, the BF-MN% was compared against the combined percentage of MN cells (alveolar macrophages, lymphocytes, and mast cells) from the manual five-part 200-cell differentiation (Additional files 5A and B). In addition, the BF-PMN% was compared against the manually obtained combined percentage of PMN cells (neutrophils and eosinophils) (Additional files 6A and B). The comparison between the automated and manual methods differentiation is depicted in Table 2.

**Fig. 4:**
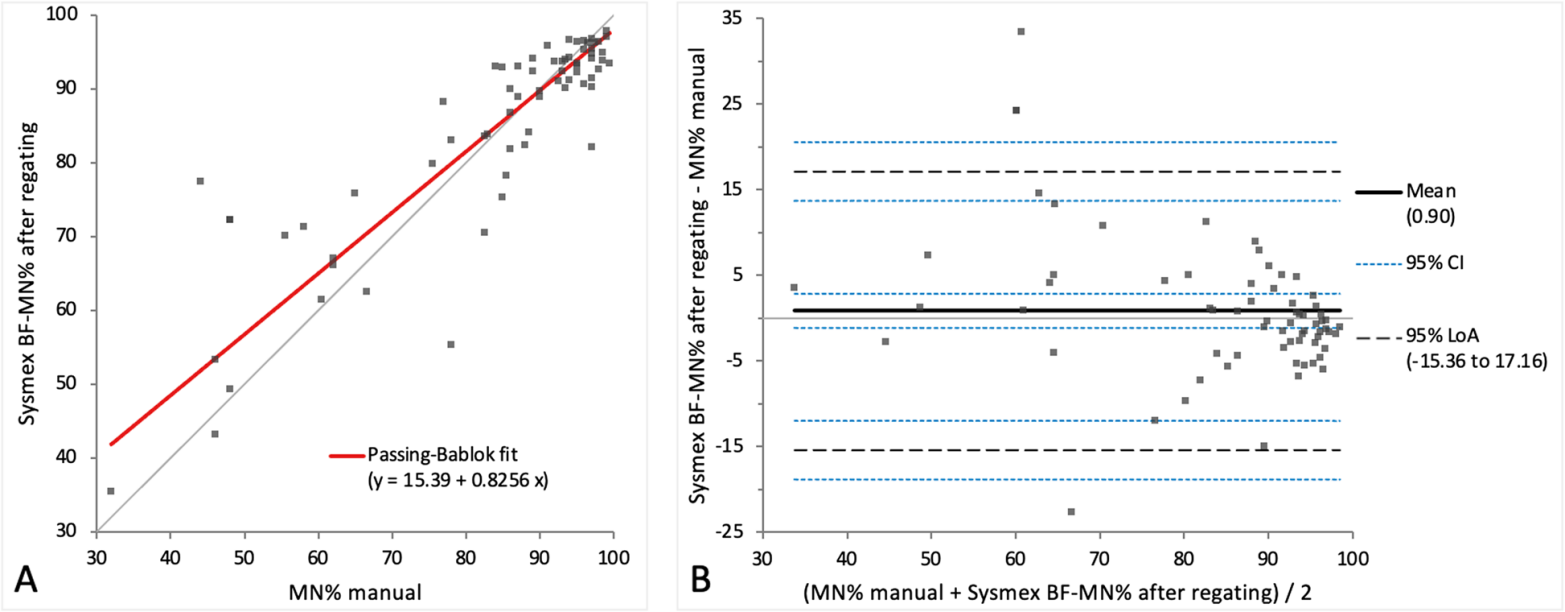
Agreement between the manual MN% and Sysmex BF-MN% after regating. The graph on the left (**A**) is a Passing-Bablok regression analysis with intercept 15.39 (4.36 to 32.68)* and slope 0.83 (0.65 to 0.94)*. The graph on the right (**B**) is a Bland–Altman difference plot. The thin horizontal grey line (0 at the y-axis) is the line of identity, and the thick black line indicates the bias (mean difference between methods), with its confidence intervals as thin blue dashed lines. The black dashed horizontal lines are the 95% limits of agreement with their 95% confidence intervals as the thin blue dashed lines. The mean difference is 0.90 (-1.09 to 2.89)* %, the Lower Limit of Agreement is -15.36 (-18.78 to -11.94)* %, the Upper Limit of Agreement is 17.16 (13.74 to 20.58)* %. *Numbers in parentheses are 95% confidence intervals

**Fig. 5:**
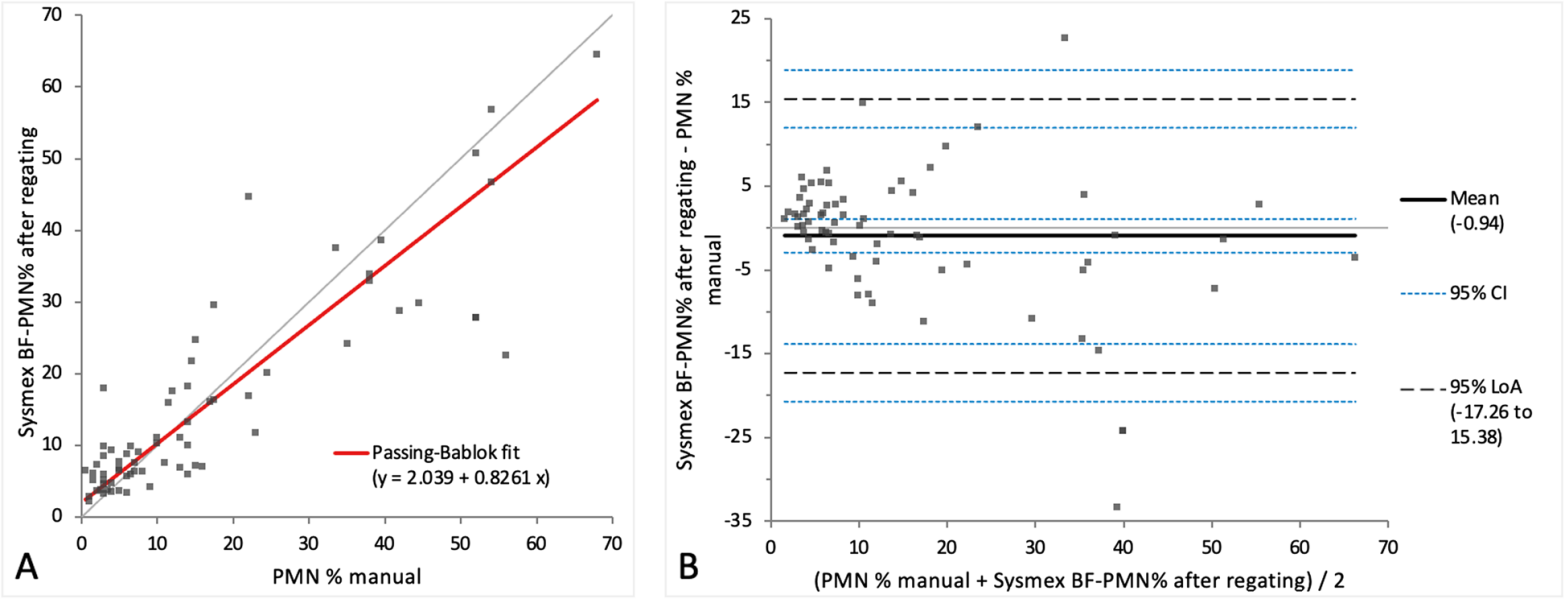
Agreement between the manual PMN% and Sysmex BF-PMN% after regating. The graph on the left (**A**) is a Passing-Bablok regression analysis with intercept 2.04 (1.06 to 3.16)* and slope 0.83 (0.66 to 0.94)*. The graph on the right (**B**) is a Bland–Altman difference plot. The thin horizontal grey line (0 at the y-axis) is the line of identity, and the thick black line indicates the bias (mean difference between methods), with its confidence intervals as thin blue dashed lines. The black dashed horizontal lines are the 95% limits of agreement with their 95% confidence intervals as the thin blue dashed lines. The mean difference is -0.94 (-2.94 to 1.06)* %, the Lower Limit of Agreement is -17.26 (-20.70 to -13.81)* %, the Upper Limit of Agreement is 15.38 (11.94 to 18.81)* %. *Numbers in parentheses are 95% confidence intervals

For the comparison study, the populations of the scattergram resulting of the Sysmex BF mode were regated manually. After regating both MN% and PMN% showed mild constant and proportional bias on Passing-Bablok regression analysis (Figs. 4A and 5A), while on Bland–Altman difference plot only very small mean difference with moderate limits of agreement was present, indicating random error (Figs. 4B and 5B). A correlation of *r* = 0.84 and 0.83 with the manual method with no statistically significant difference (*p*-value 0.92 and 0.88) between the measurements of both methods was seen.

In the majority of the samples of this study either no eosinophils were manually differentiated altogether, or their numbers were negligible. Eosinophils were noted in only 17/69 samples and their numbers ranged from 0.5 to 18% in the manual differential count via light microscopy; only in two of these cases the eosinophil percentage exceeded the PMN% cut-off of ≤ 5%. However, in case of these two samples a seemingly separate cell population of more pronounced granularity was seen extending rightwards from the usual location of PMN cell cloud representing neutrophils on the Sysmex scattergrams. This cell population, which we considered likely to be eosinophils, was not clearly separated from the usual PMN cell cloud location. However, when gated, it proved to be 17% in the case where 18% eosinophils were counted during manual differentiation via lights microscopy, while in the other case 7% were gated on Sysmex, while the manual count gave 10% (Additional file 2). Nevertheless, it must be mentioned that the current Sysmex BF mode does not offer special gating for eosinophils, as only gating in MN and PMN cells is available.

The aforementioned ACVIM Consensus Statement [5] was also applied to assess the differential cell count. Cut-off of ≤ 5% neutrophils was taken as a reference value for unremarkable BAL samples and equaled to ≤ 5 PMN% as in the majority of the samples of this study either contained no eosinophils or their numbers were negligible as explained above. The PMN% was obtained by adding percentage of neutrophils and eosinophils in the manual count and taking the percentage provided by Sysmex BF for the automated method. Thus, 57/69 (82.6%) samples could be classified as abnormal (> 5% PMN) by the Sysmex BF mode after regating and 46/69 (66.7%) by the manual technique (Table 4). The Sysmex BF mode recognized significantly more samples as abnormal than the manual method (*p*-value Fisher = 0.0495).

**Table 4:** Classification of samples by two-part differential count as unremarkable or abnormal according to the ACVIM Consensus Statement

**Method**	**Number of unremarkable samples** (≤ 5 PMN%)	**Number of abnormal samples** (> 5 PMN%)
Sysmex BF differential count after regating	12	57
Manual differential count	23	46

*BF* Body fluid, *MN* Mononuclear, *PMN* Polymorphonuclear

### Olympus VS200 slide scanner and software generated deep-learning based algorithm versus manual methods: four-part differential cell count

**Fig. 6:**
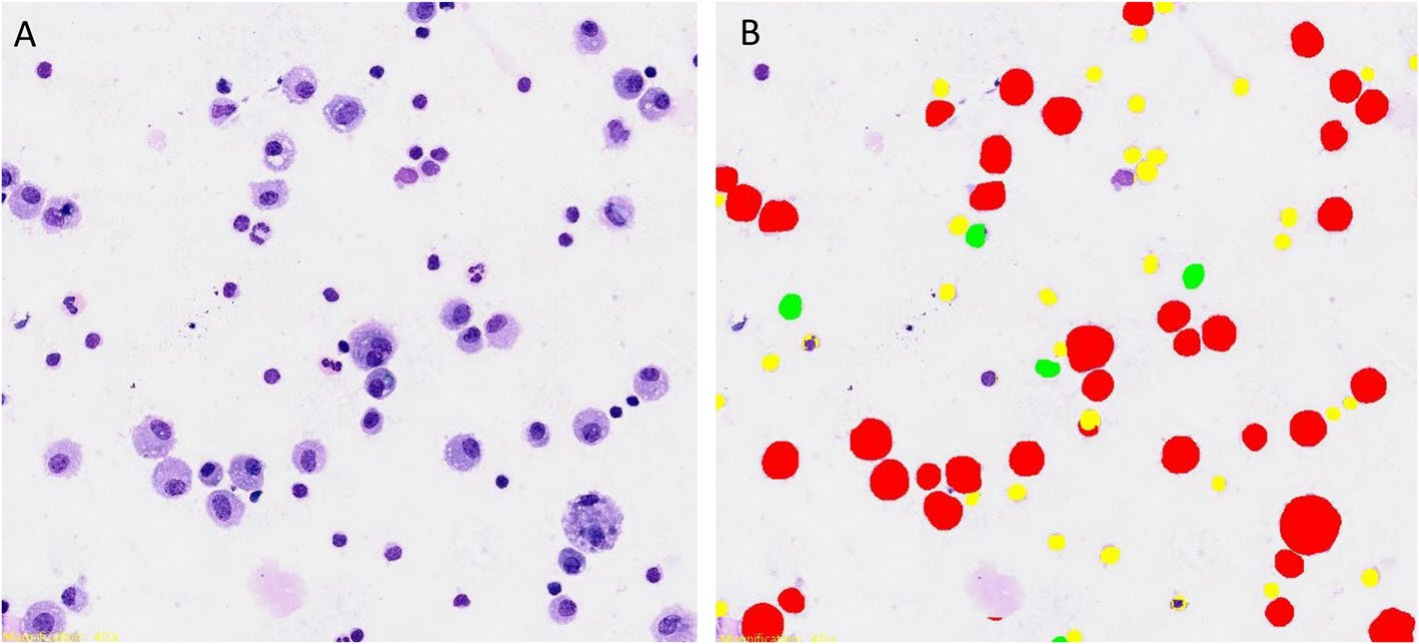
The same location on a virtual cytospin preparation slide in brightfield imaging mode. **A** native image, **B** after being analyzed with deep-learning neuronal network. Color code: red – alveolar macrophages, yellow – lymphocytes, green – neutrophils. No mast cells are shown in this figure

Using the Olympus Slideview VS200 slide scanner, two to five images from each virtual cytospin preparation slide were taken in brightfield imaging mode to reach the total number of approximately 200 cells (varying between 187 and 224 cells) per virtual slide. One cytospin preparation contained less than 200 cells and was therefore excluded from this part of the study. Of the remaining 68/69 virtual cytospin preparation slides a total of 185 digital images in well-dispersed monolayer areas were obtained. Each image was counted both manually and with the pre-developed AI algorithm obtaining a differential count in four categories: alveolar macrophages, lymphocytes, neutrophils, and mast cells (Fig. 6; for the raw data see Additional file 2). On a small number of cytospin slides a few eosinophils were also noted; however, their count was too low to appropriately train AI algorithm, therefore these cells remained unclassified; no misclassification was observed.

**Fig. 7:**
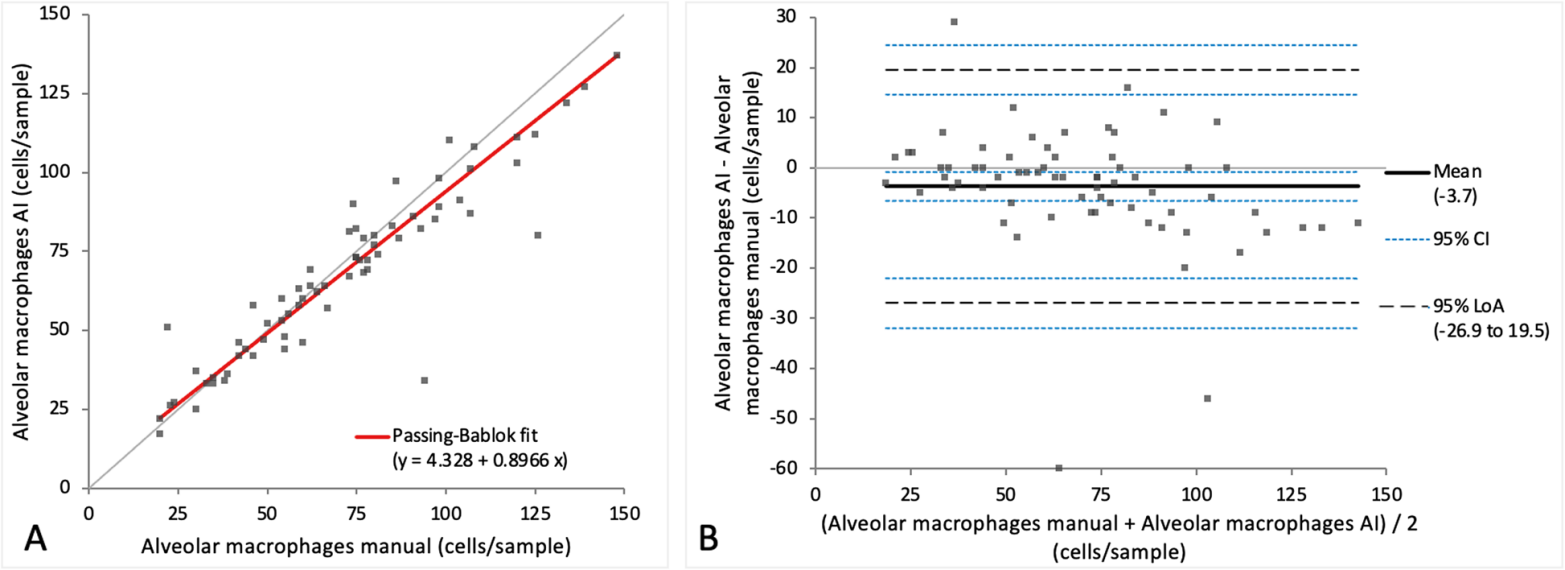
Agreement between manually and via artificial intelligence (AI) algorithm counted macrophages on images of virtual cytospin preparation slides when performing a 200-cell differential count per sample. The graph on the left (**A**) is a Passing-Bablok regression analysis with intercept 4.33 (0.72 to 7.38)* and slope 0.90 (0.85 to 0.95)*. The graph on the right (**B**) is a Bland–Altman difference plot. The thin horizontal grey line (0 at the y-axis) is the line of identity, and the thick black line indicates the bias (mean difference between methods), with its confidence intervals as thin blue dashed lines. The black dashed horizontal lines are the 95% limits of agreement with their 95% confidence intervals as the thin blue dashed lines. The mean difference is -3.7 (-6.57 to -0.84)* cells, the Lower Limit of Agreement is -26.9 (-31.85 to -22.00)* cells, the Upper Limit of Agreement is 19.5 (14.59 to 24.44)* cells. *Numbers in parentheses are 95% confidence intervals

**Fig. 8:**
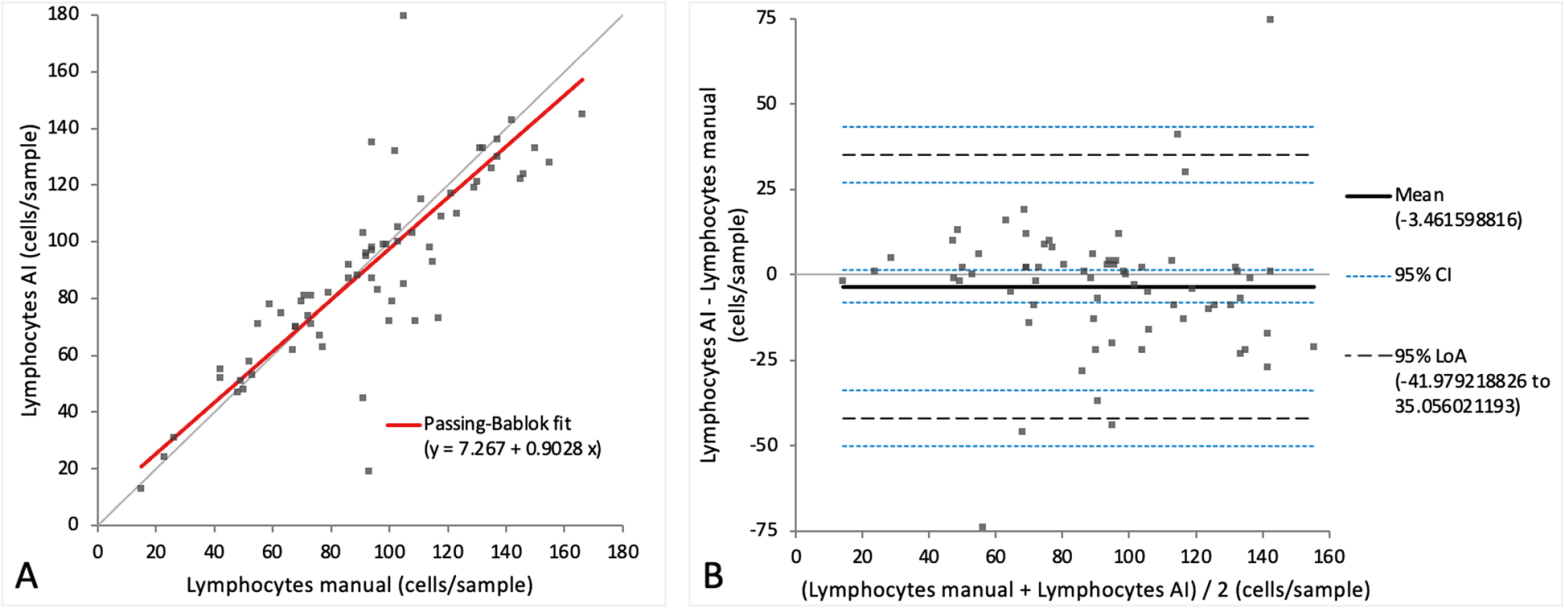
Agreement between manually and via artificial intelligence (AI) algorithm counted lymphocytes on images of virtual cytospin preparation slides when performing a 200-cell differential count per sample. The graph on the left (**A**) is a Passing-Bablok regression analysis with intercept 7.27 (-0.65 to 16.19)* and slope 0.90 (0.81 to 1.00)*. The graph on the right (**B**) is a Bland–Altman difference plot. The thin horizontal grey line (0 at the y-axis) is the line of identity, and the thick black line indicates the bias (mean difference between methods), with its confidence intervals as thin blue dashed lines. The black dashed horizontal lines are the 95% limits of agreement with their 95% confidence intervals as the thin blue dashed lines. The mean difference is -3.5 (-8.22 to 1.30)* cells, the Lower Limit of Agreement is -42.0 (-50.15 to -33.81)* cells, the Upper Limit of Agreement is 35.1 (26.89 to 43.23)* cells. *Numbers in parentheses are 95% confidence intervals

**Fig. 9:**
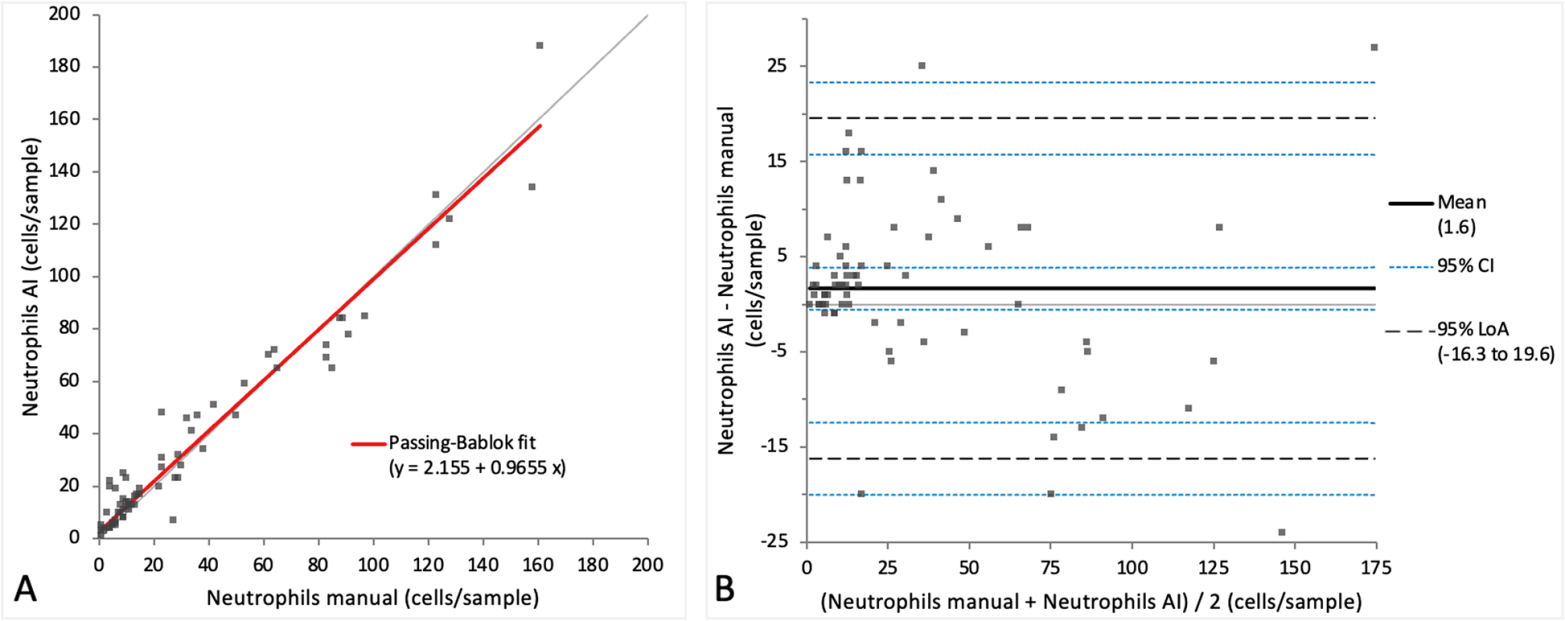
Agreement between manually and via artificial intelligence (AI) algorithm counted neutrophils on images of virtual cytospin preparation slides when performing a 200-cell differential count per sample. The graph on the left (**A**) is a Passing-Bablok regression analysis with intercept 2.2 (0.63 to 3.64)* and slope 1.0 (0.89 to 1.08)*. The graph on the right (**B**) is a Bland–Altman difference plot. The thin horizontal grey line (0 at the y-axis) is the line of identity, and the thick black line indicates the bias (mean difference between methods), with its confidence intervals as thin blue dashed lines. The black dashed horizontal lines are the 95% limits of agreement with their 95% confidence intervals as the thin blue dashed lines. The mean difference is 1.6 (-0.56 to 3.86)* cells, the Lower Limit of Agreement is -16.3 (-20.06 to -12.47)* cells, the Upper Limit of Agreement is 19.6 (15.76 to 23.36)* cells. *Numbers in parentheses are 95% confidence intervals

**Fig. 10:**
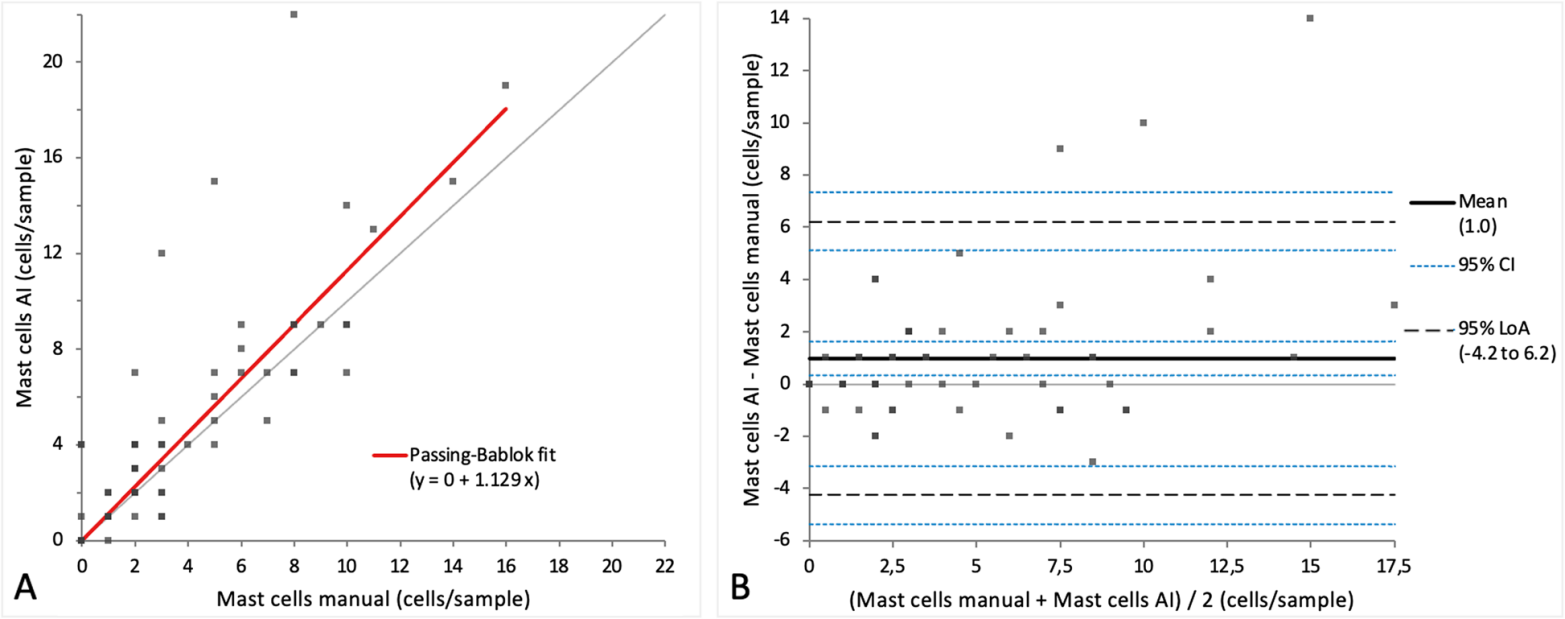
Agreement between manually and via artificial intelligence (AI) algorithm counted mast cells on images of virtual cytospin preparation slides when performing a 200-cell differential count per sample. The graph on the left (**A**) is a Passing-Bablok regression analysis with intercept 0 (-0.25 to 0.07)* and slope 1.1 (1.00 to 1.33)*. The graph on the right (**B**) is a Bland–Altman difference plot. The thin horizontal grey line (0 at the y-axis) is the line of identity, and the thick black line indicates the bias (mean difference between methods), with its confidence intervals as thin blue dashed lines. The black dashed horizontal lines are the 95% limits of agreement with their 95% confidence intervals as the thin blue dashed lines. The mean difference is 1.0 (0.34 to 1.63)* cells, the Lower Limit of Agreement is -4.2 (-5.35 to -3.14)* cells, the Upper Limit of Agreement is 6.2 (5.11 to 7.32)* cells. *Numbers in parentheses are 95% confidence intervals

When comparing the cell categories between manual differentiation and differentiation with the AI algorithm on digital slides, a very small bias was observed in Passing-Bablok regression analysis for all four cell categories (intercept varying between 0 and 7.27, while the slope varied between 0.90 and 1.1) (Figs. 7A, 8A, 9A and 10A). Very small mean difference was also observed in the Bland–Altman difference plot for all four cell categories and was as follows for each cell population: -3.7 (CI -6.57 to -0.84) cells for the alveolar macrophages, -3.5 (CI -8.22 to 1.30) cells for the lymphocytes, 1.6 (CI -0.56 to 3.86) cells for the neutrophils, and 1.0 (CI 0.34 to 1.63) cells for the mast cells (Figs. 7B, 8B, 9B and 10B). All four cell categories showed the following correlations between the methods: *r* = 0.92 for alveolar macrophages and neutrophils, *r* = 0.87 for mast cells, and *r* = 0.85 for lymphocytes. A statistically significant difference between the measurements of both methods was seen in the case of alveolar macrophages and mast cells (*p*-value 0.004 and 0.002) but not for lymphocytes and neutrophils (*p*-value 0.15 and 0.052).

**Fig. 11:**
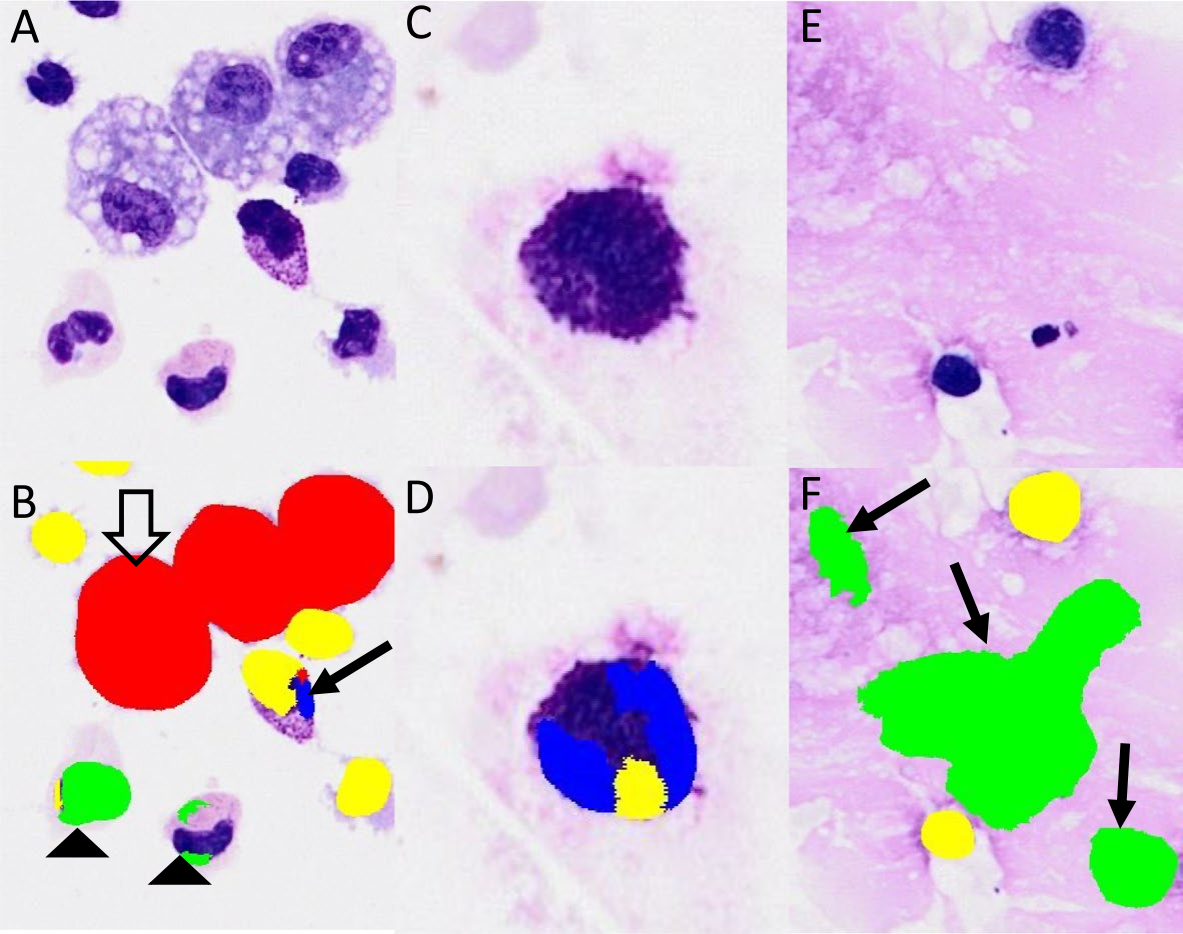
Misclassification issues with artificial intelligence (AI) algorithm. **A**, **C** and **E** native digital images from different locations on the virtual cytospin preparation slides and **B**, **D** and **F** corresponding classification performed by the AI algorithm on the same locations. **A** and **B** three alveolar macrophages counted as one (white arrow); one mast cell fragmentally classified as three cells—a lymphocyte, mast cell and an alveolar macrophage (arrow); two neutrophils fragmentally classified as four cells—three neutrophils and a lymphocyte (arrowhead), **C** and **D** one mast cell fragmentally classified as two mast cells and a lymphocyte, **E** and **F** mucus misclassified as three neutrophils (arrow). Color code: red – alveolar macrophages, green – neutrophils, yellow – lymphocytes

Although the agreement between manual differentiation and differentiation with the AI algorithm on digital slides was good, several misclassification issues of the neuronal network were observed, which explain the observed outliers. Closely located cells of the same category were often counted as one (Figs. 11A and B). This issue was more frequently observed in denser regions of the virtual cytospin preparation slides. Additionally, the recognition of some cells was fragmented, and thus one cell was counted as several cells of the same or different categories (Figs. 11A to D). Moreover, mucus in the background was often misclassified as cells, mostly neutrophils and macrophages. The presence of larger amounts of mucus on the cytospin slide proportionally gave larger amounts of “false” cells (Figs. 11E and F). The frequency and the amount of the misclassification issues varied noticeably between different samples and locations of the same cytospin preparation with their cause often not apparent to the authors. However, it seemed to be a common theme that the denser areas showed more misclassification issues.

The four-part differential cell count of the samples was also assessed by the ACVIM Consensus Statement [5], taking the cut-off of ≤ 5% for neutrophils and ≤ 2% for mast cells as a reference value for unremarkable BAL samples. Regarding the neutrophils, 49/68 (72.1%) of all samples could be classified as abnormal by AI, while manual differential cell count classified 40/68 (58.8%) of all samples as abnormal on digital images. Additionally, all samples were assessed by manual differential cell count via light microscopy and 43/69 (62.3%) of samples were classified as abnormal regarding the neutrophils. Regarding the mast cells, 26/68 (38.2%) of all samples were classified as abnormal by AI, while 23/68 (33.8%) of samples were classified so by manual differential count on digital images. Additionally, all samples were again assessed by manual differential cell count via light microscopy and 34/69 (49.3%) of samples were classified as abnormal for mast cells (Table 5). There was no significant difference in the classification according to neutrophil and mast cell counts among the three methods.

**Table 5:** Classification of samples by four-part differential count as unremarkable or abnormal according to the ACVIM Consensus Statement

**Method**	**Number of unremarkable samples** (≤ 5 neutrophils%)	**Number of abnormal samples** (> 5 neutrophils%)	**Number of unremarkable samples** (≤ 2 mast cells %)	**Number of abnormal samples** (> 2 mast cells %)
AI differential count	19	49	42	26
Manual differential count on digital images	28	40	45	23
Manual differential count via light microscopy	26	43	35	34

### Precision of the different methods and linearity of the Sysmex BF mode

The comparison of precision measurements for TNCC between the Sysmex BF mode and the manual method for samples with low, moderate, and high cell counts is depicted in Table 6. Due to the already described cell misclassification issue without regating, all Sysmex BF measurements for precision study were regated and only results after regating were analyzed further. The coefficient of variation (CV) for the BF-TNCC varied between 3.1% and 11.8%, while the CV for the manual method was between 20.6% and 62.3% and thus noticeably higher for any sample cellularity. When comparing the precision between the Sysmex BF mode and light-microscopy for the two-part differential count (Table 7), noticeably lower CV were observed for both MN% and PMN% from the Sysmex BF mode for any cellularity. Higher CVs were observed with the less cellular cell population in all three samples with both methods.

**Table 6:** Comparison of precision for TNCC between automated Sysmex BF-TNCC after regating and manual counting with hemocytometer

Method	Sample cellularity	Mean TNCC (cells × 10^6^/µL)	n	SD (cells × 10^6^/µL)	CV (%)
**Sysmex BF**	**Low**	76.8	10	3.39	4.4
	**Moderate**	263.4	10	26.34	11.8
	**High**	714.2	10	22.31	3.1
**Manual**	**Low**	42.5	10	26.48	62.3
	**Moderate**	252.5	10	49.23	19.4
	**High**	997.5	10	205.29	20.6

*BF* Body fluid, *CV* Coefficient of variation, *n* Number of replicates, *SD* Standard deviation, *TNCC* Total nucleated cell count

**Table 7:** Comparison of precision for two-part differential count between automated Sysmex BF differential after regating and manual 200-cell differentiation with light microscopy

Method	Sample cellularity	Cell type	Cell type %	n	SD (%)	CV (%)
**Sysmex BF**	**Low**^a^	MN%	81.7	10	1.61	2.0
		PMN%	18.4	10	1.35	7.3
	**Moderate**^a^	MN%	92.2	10	0.71	0.8
		PMN%	7.8	10	0.71	9.0
	**High**^a^	MN%	34.8	10	0.60	1.7
		PMN%	65.2	10	0.60	0.9
**Manual**	**Low**^a^	MN%	81.3	10	4.03	5.0
		PMN%	16.7	10	3.86	23.1
	**Moderate**^a^	MN%	94.3	10	1.64	1.7
		PMN%	5.6	10	1.71	30.6
	**High**^a^	MN%	26.1	10	6.97	26.7
		PMN%	73.9	10	6.97	9.4

*BF* Body fluid, *CV* Coefficient of variation, *MN* Mononuclear, *n* Number of replicates, *PMN* Polymorphonuclear, *SD* Standard deviation, *TNCC* Total nucleated cell count

^a^mean manual TNCC, where low = 42.5 × 10^6^ cells/µL, moderate = 252.5 × 10^6^ cells/µL, high = 997.5 × 10^6^ cells/µL

**Fig. 12:**
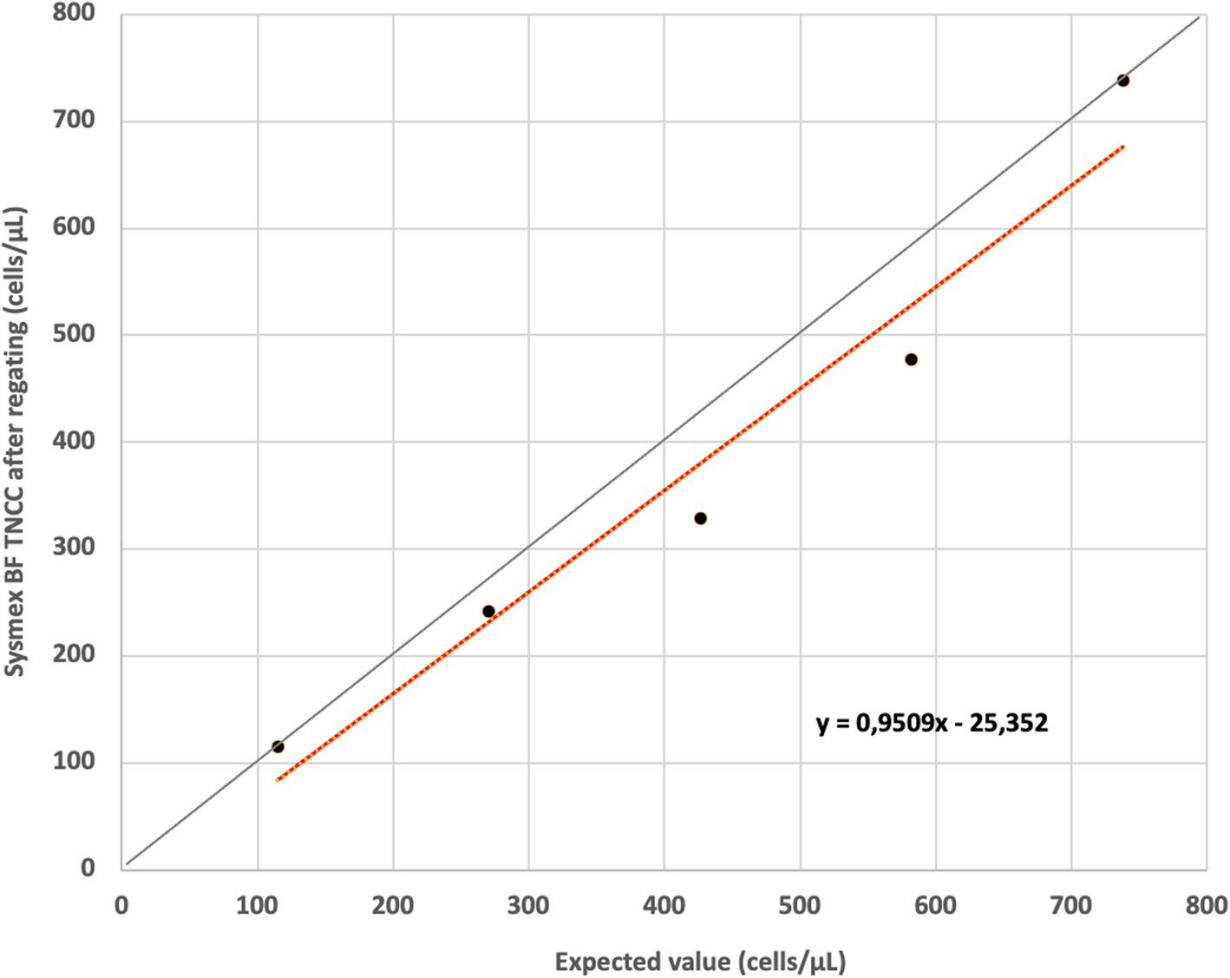
Linearity for Sysmex BF TNCC (cells/µL) after regating depicted as linear regression. The intercept is -25.35 and the slope 0.95. The grey line represents the identity line

The linearity for the Sysmex BF-TNCC after regating was good and showed recovery between 74 and 100% (Fig. 12) up to 738 cells/µL.

To assess the precision of the Olympus VS200 a precision study was undertaken for the four-part differential cell count with the same three samples for three methods and ten replicates for each sample and method: four-part 200-cell differential with Olympus VS200 AI algorithm and manually both on digital images and via light microscopy. Additionally, an 800-cell differential with the Olympus VS200 AI algorithm was also performed (Table 8). The CVs were lower for AI algorithm in all samples and all cell categories apart from alveolar macrophages in one sample with more pronounced misclassification issues, and mast cells in another sample where this cell population was of low cellularity. The CV range for the 200-cell differential count in all samples and all cell categories varied between 7.4% and 62.5%, while the CV range for 800-cell differential with AI varied between 4.7% and 24.2% and was thus lower.

**Table 8:** Precision of four-part 200-cell differential with three methods (AI algorithm and manually both on digital images and via light microscopy), as well as four-part 800-cell differential with AI algorithm

			Macrophages%				Lymphocytes %				Neutrophils %				Mast cells %			
			Manually microscope 200 cells	Manually image 200 cells	AI image 200 cells	AI image 800 cells	Manually microscope 200 cells	Manually image 200 cells	AI image 200 cells	AI image 800 cells	Manually microscope 200 cells	Manually image 200 cells	AI image 200 cells	AI image 800 cells	Manually microscope 200 cells	Manually image 200 cells	AI image 200 cells	AI image 800 cells
Sample N^o^	1	Average %	24.2	22.5	20.4	19.7	64.6	72.7	66.4	66.5	9.7	12.1	12.1	12.5	1.6	1.4	1.1	1.3
		CV%	31.4	23.8	23.6	17.7	9.9	26.8	7.4	6.0	31.9	37.3	22.1	4.7	57.8	72.0	62.5	22.7
	2	Average %	48.4	46.7	44.5	44.0	45.2	47.6	47.3	47.2	4.8	4.8	6.6	7.2	1.6	1.5	1.5	1.6
		CV%	19.8	15.6	17.5	12.8	18.4	19.4	17.2	10.5	51.8	21.3	28.2	19.6	73.1	48.7	35.2	26.2
	3	Average %	39.4	28.9	26.7	34.1	53.7	66.0	62.6	56.4	3.1	5.3	7.6	6.0	3.8	3.0	3.1	3.6
		CV%	25.5	41.0	31.4	10.5	17.4	13.7	11.4	4.7	51.8	37.9	36.4	14.2	39.7	22.3	28.6	23.0

*AI* Artificial intelligence, *CV* Coefficient of variation

## Discussion

This study compared manual and automated methods for both TNCC and differential cell counts in BAL samples from horses. Although TNCC measurements are usually performed in research only [3, 4], in the Clinic for Equine Medicine, University Animal Hospital Zurich of the Vetsuisse Faculty, University of Zurich it has been recognized that the quantification of the cellularity offers valuable information when assessing the response of the treatment to an inflammatory condition during follow-ups. This opinion is founded in the highly standardized protocol for BAL sampling utilized by a well-trained team of equine specialists/clinicians.

The results obtained with the Sysmex XN-V BF mode highlighted the importance of manual regating. Where in the case of TNCC only a few outliers showed improvement, it turned out to be essential for the differential count, improving the correlation with manual count significantly (from *r* = -0.33 to 0.84 for MN cells and from *r* = -0.32 to 0.83 for PMN cells). These improvements took place since without manually set gates, the BF mode often misclassified the PMN cells as debris, while the mononuclear cells were partly counted as MN and PMN cells. Thus, before manual regating of the Sysmex XN-V scattergrams in BF mode took place, falsely low BF-TNCC and BF-WBC, as well as inaccurate percentage distribution of BF-PMN% and BF-MN% were often reported by the respective hematology analyzer. To the best of our knowledge, no study so far has explicitly discussed the importance of regating on any hematology instrument. Indeed very few studies refer to regating in general: in one study “gating out” of cellular debris on Coulter Counter® using vital stain or cell-specific antibodies in mice has been mentioned [17], in another study of BAL in animal research for the pharmaceutical industry the disadvantage of no custom gating settings with ADVIA instrument in contrast to Sysmex hematology analyzer has been discussed and establishing of proper gating for BAL of rats and mice has been mentioned [6]. The authors also suspect that the observed correlation of *r* = -0.10 for MN and *r* = 0.01 for PMN cells in a canine CSF study with the Sysmex XN-V from 2020 could be at least partly explained through incorrect gating since in the depicted scattergrams both debris and the well-demarcated cloud of dots in the PMN cell area are of the same color [28].

Very similar values both before and after regating were obtained with BF-TNCC and BF-WBC showing almost identical correlation, mean bias, and limits of agreement in the Bland–Altman difference plot. Similar results with a correlation of *r* > 0.83 for both Sysmex BF-WBC and Sysmex BF-TNCC with the manual TNCC in pleocytic CSF samples (> 5 cells/µL) were observed in a study from 2020 [28].

A proportional bias was observed between the BF-TNCC and the manual TNCC with the automated method constantly counting more cells than the manual method. The cause of this discrepancy remains unclear. The MN and PMN cell clouds appeared clearly separated from debris in practically all samples after regating and only very few columnar epithelial cells were observed in some BAL samples thus very unlikely being the cause for the observed discrepancy. It must also be mentioned that while the manual TNCC was the reference method in this study, it can by no means be considered the gold standard and it could even be argued that BF-TNCC could be the more accurate parameter of the two since more cells are quantified at a higher precision. However, due to potential classification issues when applying the ACVIM Consensus Statement these two methods should not be used interchangeably for patient monitoring [5]. The use of automated TNCC methods can be further encouraged since adequate measurements have been obtained in some other studies for both human and animal samples with ADVIA for BAL, CSF, pleural, and ascitic fluid both with blood and CSF assay mode [6, 10, 29]; Sysmex for CSF, pleural and ascitic fluid in both blood and BF mode [22, 28, 29],  as well as Coulter Counter® for synovial fluid [30]. Automated cell count has even been deemed more accurate than manual technique in a study with ADVIA for human CSF [31].

For the two-part differential cell count a very small mean bias with narrow limits of agreement without a statistically significant difference with the manual differential cell count was observed after regating for both MN and PMN cells. The samples were classified as unremarkable or abnormal according to the ACVIM Consensus Statement [5]; more samples were classified as having increased PMN% and thus being abnormal by the Sysmex XN-V BF mode in comparison to the manual method. The clinical relevance of the cut-off value from the ACVIM Consensus statement [5], which was obtained by manual cytology, could still be debated for the present study, as different protocols for the sample collection were applied and therefore 50 mL more infusion fluid was administered in the present study. Overall, studies of various agreement between manual and automated differential counts can be found. Several studies of Sysmex analyzers in both veterinary and human medicine showed good agreement between the manual and automated two-part [21, 22] and four-part [6] differential count in both blood and BF modes in various body fluids. Meanwhile, an underestimation or overestimation of some cell populations was observed with ADVIA 120 and VetScan HM5 in blood mode for canine pleural and peritoneal fluid samples [32], while various agreement was noted for a three-part differential count with ADVIA in canine CSF when using the specific CSF assay mode [10]. Another study with Sysmex XN-V in BF mode showed negligible correlation for canine CSF samples; however, this was most likely due to incorrect gating as stated above [28]. However, the two-part differentiation has limitations for its practical applicability, since differentiation into macrophages, lymphocytes, neutrophils, eosinophils, and mast cells is required for any further clinical use and implementation of cut-off values provided by the ACVIM Consensus Statement [5]. Therefore, in future studies specific gates should be developed to separately quantify different cell populations. This goal seems to be attainable since three distinct cell populations were already clearly distinguishable on some cellular scattergrams of this study and in case of the two samples with the increased numbers of eosinophils in the manual differentiation even a more granular cell population, likely representing eosinophils, was noted on the Sysmex BF mode scattergrams. The current Sysmex BF mode, however, does not yet offer such separate gate development as only classification into MN and PMN cells is available.

Regarding precision, the Sysmex BF mode as expected outperformed the manual method for both TNCC and the two-part differential cell count in samples of various cellularity. The linearity for TNCC was also acceptable.

The four-part differential count with AI correlated with the manual differentiation in all four assessed cell categories. This study proved that AI can accurately differentiate alveolar macrophages, lymphocytes, neutrophils, and mast cells on virtual BAL cytospin preparations using the developed algorithm. When evaluating the samples with cut-offs for neutrophils and mast cells set by the ACVIM Consensus Statement [5], AI showed good agreement with the manual differential cell count via light microscopy. The observed differences between the three methods were most likely related to imprecision, as well as misclassification issues in the case of AI. While no other studies about AI differential count are available for BAL, in a study of bovine uterine cytobrush samples adequate agreement between the AI and the manual method were obtained for > 5% and > 10% PMN cell threshold with only weak agreement for > 1% cut-off. These observations are similar to our study where the best correlations were seen in the most numerous cell populations. Intra-method repeatability was also substantial in the mentioned study [27]. When thinking of future for AI algorithms in general one of their main advantages is the ability to rapidly count and differentiate thousands of cells without human bias or assistance with better precision than the manual methods. An improved algorithm, which could also recognize all the important elements of a complete BAL cytological assessment such as other cell types, hemosiderin, bacteria and so on, could spare unimaginable amount of time and effort. Technical staff with knowledge of either cell-recognition or information technologies would not needed either, as long as medical validation would be provided in a subsequent step by a person with the appropriate medical knowledge. Nevertheless, as seen in this study this method also has some drawbacks: the equipment is expensive; the development of an AI neuronal network algorithm is time-consuming and requires good quality samples containing all of the needed cell populations, and misclassification still happens. In this study the main misclassification issues were counting several closely located and touching cells as one, misclassifying mucus as cells, and fragmentally classifying one cell as several. For the first two issues, thinner cytospin preparations might be a solution. The cytospin preparation protocol could be adjusted accordingly and researched in future studies. All in all, the method is very promising and holds immense potential.

The precision of the four-part 200-cell differential count with AI algorithm outperformed the manual differentiation both via light microscopy and on digital images in all three samples for lymphocytes, two of three samples for neutrophils, and one of three for mast cells. Nevertheless, while a 200-cell differential count was used in our study, many laboratories perform a 300- or even 400-cell differential count instead to obtain better precision [1, 3]. For that reason, a four-part 800-cell differential count of the AI algorithm was also explored, which outperformed 200-cell precision not only for AI but also for both manual methods. In case of the 800-cell differential count the observed drawbacks were a slightly longer software processing time and less opportunities to choose only monolayer areas where the cells were not touching each other or overlapping. These results prove the potential of AI to improve poorly precise manual laboratory methods and are in line with a previous study of bovine uterine cytobrush samples [27].

The main limitation of this study are the highly standardized conditions under which the algorithm was developed which would preclude the successful implementation of this neuronal network if the visual appearance of the cells were even slightly altered. Due to this reason any changes in the sample preparation protocol, including a different stain or staining procedure may result in the inapplicability of the developed algorithm. Further multicenter studies are needed to broaden the algorithm including different stains and sample preparation protocols. Adding additional cell types such as erythrocytes, eosinophils, hemosiderophages, ciliated columnar epithelial cells, and goblet cells, as well as such elements as mucus and Curschmann’s spirals is also needed. The algorithm could be further optimized by setting the cell size as one of the cell recognition criteria and thus avoiding misclassification of several nearby located cells as one. In the current study, however, no further optimization of the algorithm, once the similarity had reached 0.78, could be achieved, as the similarity failed to improve upon increasing the number of training images. Predetermination of the cell size as a recognition criteria was also not possible since such function was not supported by the Olympus VS200 software used in this study.

## Conclusions

This study demonstrated that the Sysmex XN-V BF mode can be used for TNCC and two-part differential count measurements in equine BAL samples after reanalyzing the samples with manually set gates. The Olympus VS200 software can be operated without specific computer programmer skills to generate an AI algorithm. The AI four-part differential cell count correlates well with the manual methods on scanned slides, while offering a better precision than the manual methods on either scanned slides or cytospin preparations viewed by light microscopy. Therefore, AI algorithm can also be used to assess equine BAL samples. In the future multicentric studies should be undertaken to broaden the algorithm including further cell types and different preparation protocols.

## Methods

### Study design

The prospective study was conducted between July 2020 and April 2022 in the Clinical Laboratory of the Vetsuisse Faculty, University of Zurich (Switzerland). All analyses were performed using left-over material of fresh daily routine diagnostic samples, therefore no approval from the Veterinary Office of the Canton of Zurich was needed. No additional samples or volumes were collected for this study. Samples were submitted for routine diagnostic purposes by veterinarians from the Clinic for Equine Medicine, University Animal Hospital Zurich of the Vetsuisse Faculty, University of Zurich. All samples were collected by the attending veterinarian in plain tubes after 280 mL of 0.9% NaCl admixed with 20 mL 2% lidocaine had been infused into the lungs and then re-aspirated from the examined horses following a defined protocol. Measurements of TNCC, as well as preparation of the cytospin preparations, including the staining, were performed within 12 h after sampling and assessed on the same day. The scanning of the archived cytospin preparations, as well as the assessment of the digital images via AI, was done later for all slides of this study at once. For the comparison study (Table 1), TNCC and two-part differential cell count as determined by the Sysmex XN-V BF mode were compared with the results from manual techniques. Secondly, cytospin preparation slides were scanned and processed with the Olympus Slideview VS200 slide scanner and afterwards, the four-part differential cell count was performed on digital images both manually and using the VS200 software with an AI neuronal network algorithm; moreover, the cytospin slides were also manually differentiated via light microscopy (Table 1). Prior to the start of the comparison study, an AI neuronal algorithm was developed via training. Moreover, the gating of the scattergrams obtained by the Sysmex XN-V BF mode was manually optimized for equine BAL samples and all samples of the comparison study were reanalyzed using the new gates (see results section). In total 69 BAL routine samples from 58 horses presenting for either clinical workup or follow-up due to various respiratory disorders were included in the comparison study. All manual differential cell counts both via light microscopy and on digital cytospin images, as well as training of AI neuronal network algorithm were performed by one of the authors, a resident of clinical pathology (SL). All the methods were performed in accordance with relevant guidelines and regulations.

### Training of an AI neuronal network algorithm

To develop the AI neuronal network, cytospin preparations of 23 arbitrarily chosen equine routine BAL samples with morphologically well-preserved cells in light microscopy were scanned with the Olympus Slideview VS200 slide scanner (Olympus, Shinjuku, Japan) in 40 × magnification (oil immersion) to obtain virtual slides. These were processed with the VS200 software taking 46 digital images in brightfield imaging mode from monolayer areas with the best cytological quality. All cells on all images were manually labeled as either alveolar macrophages, lymphocytes, neutrophils, or mast cells. The total cell count on each image varied between 22 and 138 cells, in total reaching 3102 cells on all digital images, the cell type distribution was as follows: 1280 alveolar macrophages, 1198 lymphocytes, 529 neutrophils, and 95 mast cells. To establish a neuronal network algorithm, several configurations offered by the VS200 software were applied. The maximum similarity of 0.78 was reached with multi-label classification and specific network (RGB) after 250,000 iterations. Increasing the number of training images did not further improve the similarity at this point. This AI neuronal network algorithm was further used in this study.

### TNCC

The manual TNCC counted with a hemacytometer was compared against TNCC obtained with the automated hematology analyzer Sysmex XN1000-V (Sysmex Corporation, Kobe, Japan) using software version 3.04–00 in BF mode (Table 1). The automated measurements with Sysmex XN-V, as well as manual TNCC were performed from a 10 mL aliquot tube which had been thoroughly mixed on the nutating laboratory tube mixer (VWR international, Radnor, USA) for 5 min.

#### Manual TNCC

The manual TNCC was obtained as follows: the Neubauer improved hemacytometer (Brand, Wertheim, Germany) was filled with the sample diluted with Leucoplate (Sobioda, Montbonnot-Saint-Martin, France) or Thrombocount (Servoprax, Wesel, Germany) solution in a ratio ranging from 1:10 to 1:100 depending on the expected cellularity. The Thrombocount solution was chosen as a replacement for the Leucoplate solution as the latter was discontinued during this study and both solutions function in a similar way. The four large squares on both sides of the hemocytometer were counted for nucleated cells excluding epithelial cells, which were clearly distinguishable due to their morphology. The average number of cells per side was determined and calculations with the dilution factor were performed to obtain the number of cells per µL (cells/µL).

#### Automated TNCC count with Sysmex XN-V

The Sysmex XN-V uses fluorescence flow cytometry, and the results are reported as cells/µL. Quality Control (QC) was performed daily prior to processing routine samples using the XN-CHECK 3 Level controls (Sysmex Corporation, Kobe, Japan) covering both the normal range, as well as the abnormal low and the abnormal high range of the tested parameters. Additionally, a background check was performed every time BF analysis was accessed with the acceptable BF-WBC value of 0.001 × 10^3^ cells/µL or less [21, 33].

### Two-part differential cell count

The automated Sysmex BF two-part differential count was compared with the manual two-part 200-cell differential count obtained via light microscopy (Table 1). The samples were prepared for these analyses as described above for the TNCC.

#### Manual two-part differential cell count

Cytospin preparations were prepared as follows: 0.5 mL of thoroughly mixed BAL fluid was pipetted to three drops (corresponding to 150 µL) of 5% bovine serum albumin solution (Sigma-Aldrich, St. Louis, USA). Three drops (corresponding to 150 µL) of the obtained fluid were spun at 72xg for 10 min in a cytocentrifuge Shandon Cytospin 4 (Thermo Fisher Scientific, Waltham, USA). The cytospin preparation slides were then air dried and stained with modified Wright-Giemsa stain from Hematek Stain Pak (Siemens, Munich, Germany) on a Hematek 4488C slide stainer (Siemens, Munich, Germany). The cytospin preparation and staining process was highly standardized.

The differential count via brightfield light microscopy was performed on an Olympus BX53 microscope (Olympus, Shinjuku, Japan) in 500 × magnification. First, a manual five-part 200-cell differential cell count of alveolar macrophages, lymphocytes, neutrophils, eosinophils, and mast cells was obtained from an area of well-dispersed cells. Then the percentages of alveolar macrophages, lymphocytes, and mast cells were summed up to obtain MN%, while the percentages of neutrophils and eosinophils were summed up to obtain the PMN%. Some samples also contained very few columnar epithelial cells; however, they were not counted via light microscopy and their numbers appeared negligible in all samples containing them. It should, however, be pointed out that the BF-TNCC of Sysmex BF mode most likely still included columnar epithelial cells in its count as explained in the background section and thus was the only parameter or method of this entire article doing so.

#### Automated Sysmex BF two-part differential cell count

The Sysmex XN-V BF mode offers a two-part differential count in MN or PMN cells using fluorescence flow cytometry. The samples were processed as described above for automated TNCC. The results were reported as percentages for relative cell counts.

### Four-part differential cell count

The cytospin preparations were scanned with the Olympus Slideview VS200 slide scanner and processed with VS200 software using the herein developed AI neuronal network algorithm. Digital images of approximately 200 cells from each sample were taken. The digital images were taken from well dispersed areas chosen by the operator, where the cells were located individually and touched as little as possible. While in very cellular samples the periphery often appeared to be more suited to this purpose, in samples of moderate to low cellularity areas throughout the whole cytospin preparation slide could be chosen. If the chosen area contained less than 200 cells, an additional image with the still needed number of cells was taken in a different equally suitable area of the same cytospin preparation. Four-part 200-cell differential cell count of alveolar macrophages, lymphocytes, neutrophils, and mast cells was then performed on these digital images. Each image was counted in its entirety both manually and with the pre-developed AI algorithm, then both methods were compared. Thus, both methods were applied on exact the same image and cells. In addition, a four-part differential cell count was also performed on each cytospin preparation via light microscopy. Some samples also contained very few columnar epithelial cells; however, they were not counted by none of the methods and their numbers appeared negligible in all of these samples.

### Precision of the different methods and linearity of the Sysmex BF mode

The precision was tested for both TNCC and differential cell counts with all counting methods performed. Precision for TNCC and the two-part differential count was assessed in samples with low, moderate, and high cell count with manual methods and the Sysmex XN-V analyzer BF mode. The precision for the four-part differential cell count was assessed in three BAL samples by performing 200-differential cell count in well-dispersed areas with three methods: manually via light microscopy on cytospin preparations; manually on digital images and with AI algorithm on digital images. To test AI precision with more cells counted, 800-cell differentiations were performed on digital images of the same three samples using the AI algorithm. All precision experiments were performed by repeating measurements 8 to 10 times within run, using subset of both the 69 samples from the comparison study and other routine samples not part of the comparative study.

Linearity of TNCC obtained with Sysmex BF was measured within-run with five dilutions (20%, 40%, 60%, 80%, 100%) from a routine sample (BF-TNCC after regating 735 cells/µL).

### Statistical analysis

Passing-Bablok regression and Bland–Altman difference plot were used to compare the methods and assess the bias. Spearman`s rank correlation coefficient (r) was used to determine the correlation between different methods. For the assessment of precision, standard deviation (SD) and coefficient of variation (CV) were calculated. Linear regression was applied for the evaluation of linearity. Wilcoxon signed-rank test was used to compare the measurements of the same parameter in the same sample. Frequencies were compared using the Fisher’s exact test for two groups and the Chi-square test for three goups. A *p*-value < 0.05 was considered statistically significant. The statistical analysis was performed with Analyse-it on Microsoft Excel version 2108 (Build 14326.20404) with the exception of the Fisher’s exact and the chi-square tests, which were performed using GraphPad Prism (GraphPad Software, Boston, MA, USA; Version 9).

### Supplementary Information

**Additional file 1. **The manually set gates as seen on the regular (A) and the extended (B) scattergram of Sysmex XN-V BF mode. Debris is depicted as dark blue, MN cells as green, and PMN cells as light blue.

**Additional file 2. **Raw data for all performed cell counts of all cell types. The cytospin preparation of the sample Nr 6 contained less than 200 cells and was therefore excluded from the four-part differential cell count.

**Additional file 3. **Agreement between the manual TNCC and Sysmex BF-TNCC (cells/µL) before regating. The graph on the left (A) is a Passing-Bablok regression analysis with intercept 58.00 (-8.66 to 107.80)* and slope 1.55 (1.23 to 1.88)*. The graph on the right (B) is a Bland-Altman difference plot. The thin horizontal grey line (0 to the y-axis) is the line of identity, and the thick line black line indicates the bias (mean difference between moods), with its confidence intervals as thin blue dashes lines. The black dashes horizontal lines are the 95% limits of agreement with their 95% confidence intervals as the thin blue dashes lines. The mean difference is 171.3 (136.44 to 206.23)* (cells/µL), the Lower Limit of Agreement is 113.4 (-173.32 to -53.47)* cells/µL, the Upper Limit of Agreement is 456.1 (396.13 to 515.99)* cells/µL. *Numbers in parentheses are 95% confidence intervals.

**Additional file 4. **Agreement between the manual TNCC and Sysmex BF-WBC (cells/µL) before regating. The graph on the left (A) is a Passing-Bablock regression analysis with intercept 39.00 (-19.75 to 93.00)* and slope 1.48 (1.17 to 1.83)*. The graph on the right (B) is a Bland-Altman difference plot. The thin horizontal grey line (0 at the y-axis) is the line of identity, and the thick black line indicates the bias (mean difference between methods), with its confidence intervals as thin blue dashes lines. The black dashed horizontal lines are the 95% limits of agreement with their 95% limits of agreement with their 95% confidence intervals as the thin blue dashed line. The mean difference is 138.5 (102.22 to 174.82)* cells/µL, the Lower Limit of Agreement is -157.6 (-219.97 to -95.30)* cells/µL, the Upper Limit of Agreement is 434.7 (372.34 to 497.01)* cells/µL. *Number in parentheses are 95% confidence intervals.

**Additional file 5. **Agreement between the manual MN% and Sysmex BF-MN% before regating. The graph on th left (A) is a Passing-Bablok regression analysis, no linear equation can be calculated. The graph on the right (B) is a Bland-Altman difference plot. The thin horizontal grey line (0 at the y-axis) is the line of identity, and the thick black line indicates the bias (mean difference between methods), with its confidence intervals as this blue dashed lines. The black dashed horizontal lines are the 95% limits of agreement with their 95% confidence intervals as the thin blue dashed lines. The mean difference is -48.05 (-54.31 to -41.79)* %, the Lower Limit of Agreement is -99.10 (-109.84 to -88.35)* %, the Upper Limit of Agreement is 3.00 (-7.74 to 13.75)* %. *Numbers in parentheses are 95% confidence intervals.

**Additional file 6. **Agreement between the manual PMN% and Sysmex BF-PMN% before regating. The graph on the left (A) is a Passing-Bablok regression analyses, no linear equation can be calculated. The graph on the right (B) is a Bland-Altman difference plot. The thin horizontal grey line (0 at the y-axis) is the line of identity, and the thick black line indicates the bias (mean difference between methods), with its confidence intervals as thin blue dashed lines. The black dashed horizontal lines are the 95% limits of agreement with their 95% confidence intervals as the thin blue dashed lines. The mean difference is 48. 00 (41.76 to 54.25)* %, the Lower Limit of Agreements is -2.98 (-13.71 to 7.75)* %, the Upper Limit of Agreement is 98.99 (88.26 to 109.72)* %. * Numbers in parentheses are 95% confidence intervals.

## Data Availability

The datasets used and/or analyzed during the current study are available from the corresponding author on reasonable request.

## Abbreviations

ACVIM: American College of Veterinary Internal Medicine
AI: Artificial intelligence
BAL: Bronchoalveolar lavage
BF: Body fluid
cells/µL: Cells per microliter
CI: Confidence interval
CSF: Cerebrospinal fluid
CV: Coefficient of variation
MN: Mononuclear
n: Number of replicates
PMN: Polymorphonuclear
QC: Quality control
r: Spearman’s rank correlation coefficient
SD: Standard deviation
TNCC: Total nucleated cell count
WBC: White blood cell
