# Supplementary material for: Comparison of Sysmex XN-V body fluid mode and deep-learning-based quantification with manual techniques for total nucleated cell count and differential count for equine bronchoalveolar lavage samples
Source: BMC Vet Res. 2024 Feb 5;20:48. doi: 10.1186/s12917-024-03884-5 (PMC10840287; doi:10.1186/s12917-024-03884-5)
Supplement: Supplementary file 1 — Additional file 1. The manually set gates as seen on the regular (A) and the extended (B) scattergram of Sysmex XN-V BF mode. Debris is depicted as dark blue, MN cells as green, and PMN cells as light blue. [file 12917_2024_3884_MOESM1_ESM.pdf]

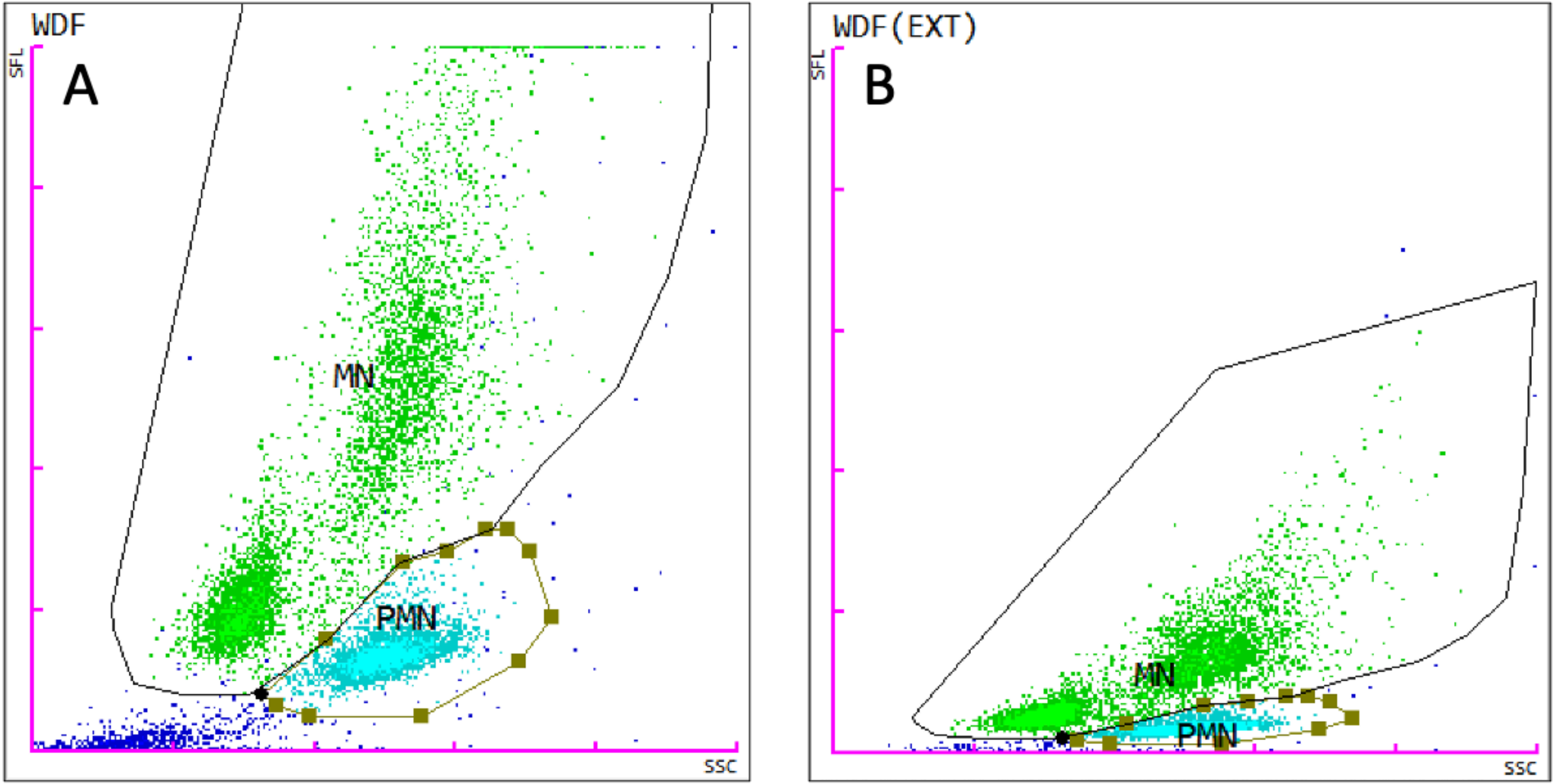

Additional file 1. The manually set gates as seen on the regular (A) and the extended (B) scattergram of Sysmex XN-V BF mode. Debris is depicted as dark blue, MN cells as green, and PMN cells as light blue.
