# Supplementary material for: Comparison of Sysmex XN-V body fluid mode and deep-learning-based quantification with manual techniques for total nucleated cell count and differential count for equine bronchoalveolar lavage samples
Source: BMC Vet Res. 2024 Feb 5;20:48. doi: 10.1186/s12917-024-03884-5 (PMC10840287; doi:10.1186/s12917-024-03884-5)
Supplement: Supplementary file 4 — Additional file 4. Agreement between the manual TNCC and Sysmex BF-WBC (cells/µL) before regating. The graph on the left (A) is a Passing-Bablock regression analysis with intercept 39.00 (-19.75 to 93.00)* and slope 1.48 (1.17 to 1.83)*. The graph on the right (B) is a Bland-Altman difference plot. The thin horizontal grey line (0 at the y-axis) is the line of identity, and the thick black line indicates the bias (mean difference between methods), with its confidence intervals as thin blue dashes lines. The black dashed horizontal lines are the 95% limits of agreement with their 95% limits of agreement with their 95% confidence intervals as the thin blue dashed line. The mean difference is 138.5 (102.22 to 174.82)* cells/µL, the Lower Limit of Agreement is -157.6 (-219.97 to -95.30)* cells/µL, the Upper Limit of Agreement is 434.7 (372.34 to 497.01)* cells/µL. *Number in parentheses are 95% confidence intervals. [file 12917_2024_3884_MOESM4_ESM.pdf]

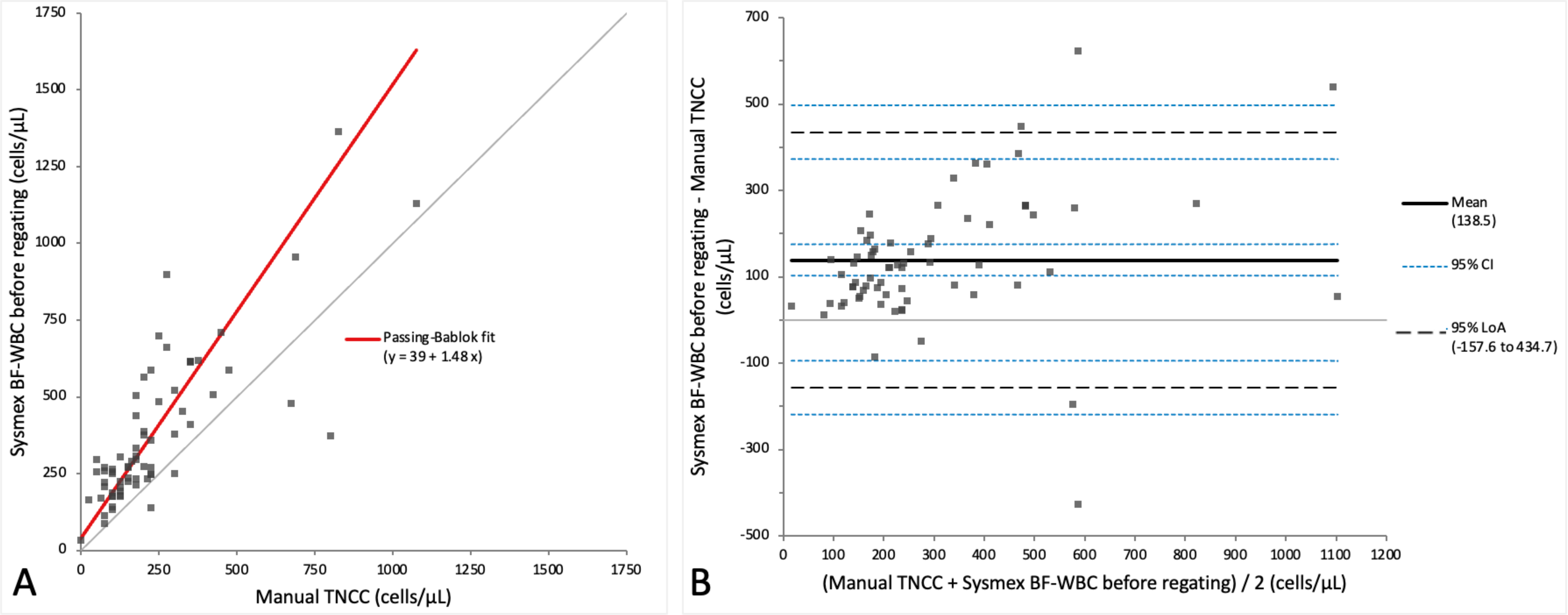

Additional file 4. Agreement between the manual TNCC and Sysmex BF-WBC (cells/µL) before regating. The graph on the left (A) is a Passing-Bablok regression analysis with intercept 39.00 (-19.75 to 93.00)* and slope 1.48 (1.17 to 1.83)*. The graph on the right (B) is a Bland-Altman difference plot. The thin horizontal grey line (0 at the y-axis) is the line of identity, and the thick black line indicates the bias (mean difference between methods), with its confidence intervals as thin blue dashed lines. The black dashed horizontal lines are the 95% limits of agreement with their 95% confidence intervals as the thin blue dashed lines. The mean difference is 138.5 (102.22 to 174.82)* cells/µL, the Lower Limit of Agreement is -157.6 (-219.97 to -95.30)* cells/µL, the Upper Limit of Agreement is 434.7 (372.34 to 497.01)* cells/µL.

*Numbers in parentheses are 95% confidence intervals.
